# Bibliometric analysis of dietary patterns in Parkinson’s disease: from Mediterranean diet to emerging trends

**DOI:** 10.3389/fnagi.2026.1954819

**Published:** 2026-09-23

**Authors:** Ting Yu, Tong Nie, Qingxin Mai, Lin Wang, Zuyan Hu, Mingjie Wu, Mingxia Pan, Yongqi Liang, Hanjiao Liu

**Affiliations:** 1 Shenzhen Hospital of Integrated Traditional Chinese and Western Medicine, Shenzhen, Guangdong, China; 2 School of Nursing, Fujian University of Traditional Chinese Medicine, Fuzhou, Fujian, China; 3 Fengcheng People’s Hospital, Fengcheng, Jiangxi, China; 4 Shenzhen Clinical College of Integrated Traditional Chinese and Western Medicine, Affiliated to Guangzhou University of Traditional Chinese Medicine, Shenzhen, Guangdong, China

**Keywords:** bibliometric analysis, dietary patterns, gut-brain axis, Mediterranean diet, Parkinson’s disease

## Abstract

**Background:**

Parkinson’s disease (PD) is one of the fastest-growing neurological disorders. Treatments remain symptomatic, with no disease-modifying therapies available. Recent preclinical and epidemiological studies suggest that dietary patterns may influence PD pathology through the microbiota-gut-brain axis by modulating gut microbiota. However, evidence remains conflicting: some studies report protective effects of specific diets, while large-scale prospective studies find no significant associations. These discrepancies reflect differences in study design and dietary assessment and a lack of systematic understanding of the field. No bibliometric study has systematically analyzed this area. Therefore, this study uses bibliometric methods to map research hotspots and evolutionary trends and identify knowledge gaps.

**Methods:**

Systematic searches were conducted in the Web of Science Core Collection and Scopus databases to identify literature on dietary patterns in Parkinson’s disease from inception to June 11, 2026. CiteSpace was used to perform bibliometric analyses, construct a knowledge map, and identify research hotspots and evolutionary trends.

**Results:**

A total of 424 publications were included. The top three most productive countries were the USA, China, and Italy, and the leading institutions included the NIH, Harvard University, and Johns Hopkins University. Key authors included Mark P. Mattson, Alberto Ascherio, and Xiang Gao. Current research hotspots are mainly “Mediterranean diet”, “ketogenic diet”, “caloric restriction”, “gut microbiota”, “gut-brain axis”, and “oxidative stress”. Emerging topics include “probiotic agent”, “prebiotic agent”, “fecal microbiota transplantation”, and “mild cognitive impairment”.

**Conclusion:**

Dietary patterns may influence PD through synergistic mechanisms, with their roles in regulating oxidative stress, neuroinflammation, mitochondrial function, and gut microbiota progressively being revealed. The microbiota-gut-brain axis may represent an important pathway linking dietary factors to central nervous system function through microbial metabolites, immunomodulation, and neural signaling. Existing studies suggest potential associations between specific dietary patterns and PD risk or clinical outcomes; however, methodological heterogeneity and the predominantly observational evidence base have precluded consistent conclusions and limited clinical translation. Therefore, no specific dietary pattern can currently be recommended with confidence for the clinical management of PD. As a modifiable lifestyle factor, dietary patterns bridge metabolic dysregulation and neurodegeneration, and their integration into PD management remains a future direction.

**Systematic review registration:**

https://osf.io/vgc9p, identifier VGC9P.

## 1 Introduction

Parkinson’s disease (PD) is a chronic neurodegenerative disorder commonly affecting middle-aged and older adults, characterized by a complex array of motor and non-motor symptoms (Tanner and Ostrem, 2024). With the acceleration of global population aging, PD has become one of the fastest-growing neurological disorders worldwide. It is projected that by 2050, the total number of PD patients globally will reach 25.2 million, representing a 112% increase from 2021 (Su et al., 2025). However, all currently approved pharmacological agents are symptomatic treatments, and no disease-modifying therapy has yet been shown to halt or reverse the neurodegenerative process (Poewe et al., 2017). As the disease advances, motor complications and non-motor symptoms progressively worsen, placing an ever-increasing burden on both patients and caregivers (Chen and Zhou, 2025; Gustafsson et al., 2025). Given the difficulty of reversing disease progression with existing therapeutic approaches, exploring synergistically optimizable adjunctive management strategies has become an inevitable direction. In 2025, the International Parkinson and Movement Disorder Society (MDS) Health Working Group proposed a “Holistic Wellness Prescription” framework, which defines the core of health management as “people living with a disease can improve their quality of life on a daily basis through proactive lifestyle choices,” with key elements encompassing healthy diet, regular exercise, adequate sleep, mind-body therapies, and social connections. The framework emphasizes that these highly modifiable factors should be addressed from the time of diagnosis or even during the prodromal phase (Subramanian et al., 2026). Among these five core elements, healthy diet has garnered particular attention due to its multiple associations with PD pathophysiology. Specific dietary patterns may influence the onset and progression of PD through the regulation of oxidative stress, neuroinflammation, mitochondrial function, and the gut microbiota, thereby holding potential as adjunctive therapeutic strategies (Fekete et al., 2025; Pokora et al., 2025; Sobral et al., 2025).

Among the classical dietary patterns, the Mediterranean diet has been associated with better symptom control, slower disease progression, and reduced all-cause mortality (Zuliani et al., 2026). Protein-redistribution diets, by optimizing the temporal distribution of protein intake, can reduce competitive amino acid absorption with levodopa, thereby enhancing clinical efficacy and reducing motor fluctuations (Boelens Keun et al., 2021). In recent years, emerging dietary patterns have been continuously expanding the boundaries of dietary interventions for PD. The ketogenic diet, as a metabolic intervention strategy, has shown potential in improving non-motor symptoms such as cognitive impairment, anxiety, and quality of life in PD patients (de Farias et al., 2025; Golob et al., 2025). Plant-based diets have also shown preliminary benefits in PD. A small pilot study suggested improvements in motor performance (Baroni et al., 2011), while emerging evidence has indicated potential reductions in inflammatory markers and improvements in non-motor symptoms (Metcalfe-Roach, 2024; Tağraf et al., 2026). The neuroprotective effects of the aforementioned dietary patterns are partly mediated by the microbiota-gut-brain axis; dietary components can be metabolized by the gut microbiota to produce bioactive metabolites such as short-chain fatty acids (SCFAs), which may influence central nervous system function through immunomodulation and vagal signaling (Tuigunov et al., 2025). However, the existing evidence remains markedly fragmented and contradictory. At the level of disease prevention, greater adherence to the MIND and Mediterranean diets has been associated with later PD onset, with differences in age at onset of up to 17.4 years for the MIND diet in women and 8.4 years for the Greek Mediterranean diet in men (Metcalfe-Roach et al., 2021). In addition, high adherence to the Mediterranean diet has been associated with an approximately 21% lower probability of prodromal PD (Maraki et al., 2019). Nevertheless, Chen et al. (2026), based on two large-scale U.S. prospective cohorts, comprehensively evaluated eight healthy dietary patterns and found no significant association between any of them and PD risk. These inconsistencies arise not only from differences in study design, sample characteristics, and dietary assessment methods but also reflect a lack of systematic understanding of the overall research landscape in this field. Meanwhile, the prevalence of malnutrition risk in PD patients ranges from 3% to 60% (Sheard et al., 2011), and a Chinese population-based study reported an overall nutritional abnormality rate of 69.2% (Yang et al., 2020), further underscoring the clinical urgency of synthesizing existing evidence and optimizing dietary intervention strategies.

Bibliometrics is an important method for systematically revealing the knowledge structure of a research field, identifying research hotspots, and tracking evolutionary trends (Chen, 2004). Previous studies have largely focused on narrative reviews or meta-analyses of single dietary patterns, and no bibliometric study has yet systematically analyzed PD dietary pattern research. Therefore, the present study aims to employ CiteSpace for bibliometric analysis to reveal, for the first time, the knowledge structure, evolutionary trajectories, and frontier transitions in this field, thereby providing a reference for synthesizing existing evidence and clarifying the research landscape. This study was registered on the Open Science Framework^1^.

## 2 Materials and methods

### 2.1 Data sources and search strategy

The Web of Science Core Collection (WoSCC) and Scopus databases were systematically searched for literature on dietary patterns in PD from inception to June 11, 2026. Both databases provide broad multidisciplinary coverage and are widely used for bibliometric analysis, while their complementary coverage helps broaden the literature base beyond reliance on a single database (Mongeon and Paul-Hus, 2016; AlRyalat et al., 2019). Taking WoSCC as an example, the search strategy was formulated as follows: (TS = (“Parkinson” OR “Parkinson’s disease”)) AND TS = (“Mediterranean diet” OR “ketogenic diet” OR “MIND diet” OR “plant-based diet” OR “vegetarian diet” OR “vegan diet” OR “DASH diet” OR “low-protein diet” OR “intermittent fasting” OR “low-fat diet” OR “caloric restriction” OR “Paleolithic diet” OR “paleo diet” OR “dietary pattern” OR “diet* intervention” OR “whole diet” OR “nutrition* pattern*”). The detailed search strategies are provided in the Supplementary material.

### 2.2 Inclusion and exclusion criteria

Based on the research objectives, the inclusion and exclusion criteria were established as follows. Inclusion criteria: (1) peer-reviewed literature of the article or review type; (2) studies focusing on the application or association of dietary patterns in PD, including but not limited to the Mediterranean diet, ketogenic diet, MIND diet, and other dietary patterns, covering clinical studies, preclinical studies, mechanistic studies, epidemiological investigations, and systematic reviews; (3) publications in English. Exclusion criteria: (1) literature types including conference abstracts, editorials, letters, news items, comments, corrections, book chapters, theses, and duplicate publications; (2) studies irrelevant to the present topic (e.g., those discussing only single nutrients, vitamins, or plant extracts rather than overall dietary patterns), duplicate publications, or articles for which full texts could not be accessed.

### 2.3 Literature screening and data standardization

The retrieved bibliographic records were imported into EndNote software for duplicate removal. Two researchers independently screened the literature according to the inclusion and exclusion criteria described above, and disagreements were resolved through discussion and consultation. After the final eligible publications were identified, the corresponding records were exported from EndNote in RIS format and converted using CiteSpace’s built-in data conversion function. The converted records were subsequently used for bibliometric analysis. At the country/region level, “England,” “Scotland,” “Wales,” and “North Ireland” were merged into “UK”; “USA” and “United States of America” were merged into “USA”; and “Peoples R China” was standardized as “China.” At the institutional level, NIH-affiliated institutes such as NIA and NINDS were unified as “National Institutes of Health (NIH)”; Harvard-affiliated institutions including “Harvard Medical School” and “Massachusetts General Hospital” were merged into “Harvard University”; and “Johns Hopkins Medicine” and related variants were consolidated as “Johns Hopkins University.” At the terminology level, “Parkinson disease” was unified as “Parkinson’s Disease”; “Alzheimer’s disease” and “Alzheimers disease” were standardized as “Alzheimer’s disease”; and “gut microbiome” and “gut microbiota” were merged into “gut microbiota.” At the keyword level, “Mediterranean diet” and “Mediterranean dietary pattern” were merged into “Mediterranean diet,” and “caloric restriction” and “energy restriction” were merged into “caloric restriction.” These preprocessing steps effectively avoided node redundancy caused by synonymous expressions and enhanced the accuracy and reliability of the cluster analyses.

### 2.4 Data analysis

Annual publication data were imported into Excel 2021 for statistical analysis and line chart generation. Bibliometric analyses were performed using CiteSpace 6.4 R1 software. The time span was set from 2000 to 2026, with 1-year time slices. The g-index (*k* = 25) was adopted as the selection criterion, with Top N set to 50 or Top N% set to 10%, and the Pathfinder pruning algorithm was applied. Based on these parameters, collaboration networks of countries, institutions, and authors were constructed to reveal the distribution of research forces; keyword co-occurrence networks, cluster maps, burst detection, and timezone views were generated to identify research hotspots and their dynamic evolution; and co-citation networks and timeline views of cited references were also constructed to uncover the intellectual base and frontier transitions. Together, these analyses comprehensively delineated the dynamic evolutionary characteristics, knowledge structure, hotspot shifts, and emerging trends in PD dietary pattern research. In the generated networks, nodes represent analytical units such as keywords, countries, institutions, or authors. Node size reflects frequency, color indicates temporal evolution or cluster membership, and connecting lines denote co-occurrence, collaboration, or co-citation relationships. Betweenness centrality serves as a key indicator of the bridging role of a node within the network, and is calculated using the following formula:


$$g\,\left(v\right)=\underset{s\neq v\neq t}{\sum }\frac{\sigma_{s\,t}\,\left(v\right)}{\sigma_{s\,t}}$$

where σ_*st*_(*v*) is the total number of shortest paths from node s to node t, and σ_*st*_(*v*) is the number of those paths that pass through node v. Nodes with a centrality ≥0.1 are generally considered to play critical bridging roles across different research themes. Accordingly, in the present study, nodes with a betweenness centrality ≥0.1 were marked with purple rings in the CiteSpace-generated maps to highlight their bridging positions in knowledge flow.

## 3 Results

A total of 1,797 articles were initially identified from the WOSCC and Scopus databases; after deduplication and screening, 424 were ultimately included, as shown in Figure 1.

**FIGURE 1:**
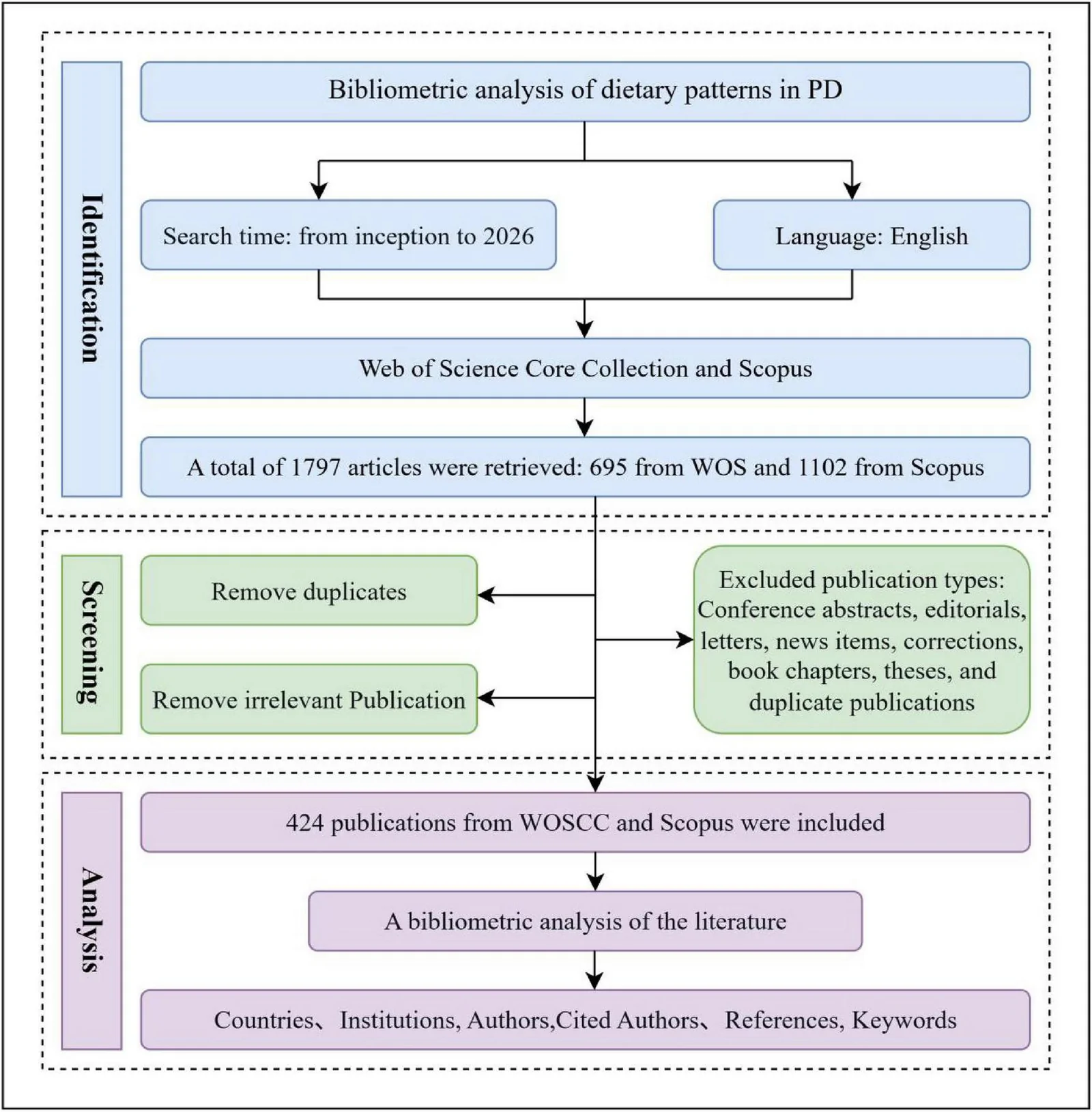
Flowchart of the literature screening process.

### 3.1 Annual publication output

The annual publication output in the field of PD dietary pattern research showed a fluctuating upward trend (Figure 2), with a cumulative total of 424 publications. The period from 2000 to 2016 represented the nascent stage of research, with an average of approximately 4 publications per year. Early studies predominantly focused on caloric restriction and neuroprotective mechanisms, exploring the potential protective effects of dietary interventions against neurodegeneration. The period from 2017 to 2021 marked a stage of initial growth, with an average of approximately 20 publications per year. During this phase, research gradually expanded toward metabolic mechanisms, with increasing exploration of metabolic intervention strategies such as the ketogenic diet and intermittent fasting, while the association between classical dietary patterns, particularly the Mediterranean diet, and PD risk continued to attract attention. The period from 2022 to 2026 represented a phase of rapid growth, with a marked increase in annual publication output and a peak of 79 publications in 2025. Research perspectives deepened from macroscopic dietary patterns toward the microbiota-gut-brain axis, with growing interest in the role of gut microbiota and their metabolites in neuroinflammation regulation, alongside emerging exploration of novel intervention modalities such as probiotics, prebiotics, and fecal microbiota transplantation. It should be noted that the 2026 data included only the first half of the year, and thus the annual publication count and trend interpretation for this year should be approached with caution.

**FIGURE 2:**
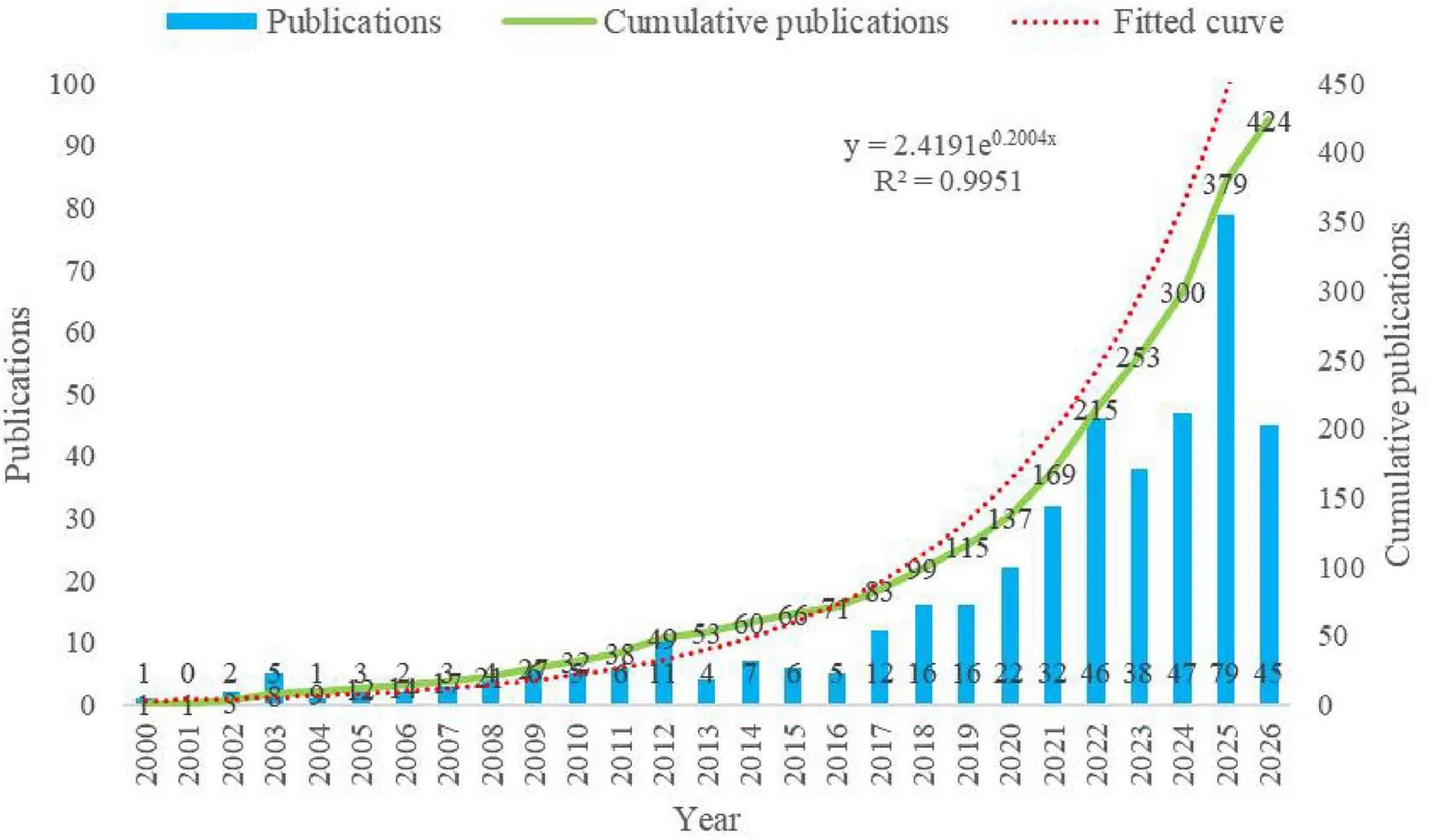
Annual publication trends in dietary pattern research for PD.

### 3.2 Country and institution collaboration networks

Collaboration networks were constructed using “countries” and “institutions” as nodes to visually present their collaborative relationships. The country collaboration network (Figure 3a) comprised 48 nodes (*N* = 48) and 98 collaborative links (*E* = 98), with a network density of 0.0869. The largest connected component included 34 countries (70%), indicating that countries have established relatively close collaborative foundations in this field. The top 10 most productive countries are presented in Table 1. The USA (70 publications), China (45 publications), and Italy (21 publications) were the top three most productive countries, among which the USA (betweenness centrality = 0.35) and Italy (betweenness centrality = 0.23) exhibited purple rings, indicating that these two countries are not only core productive nations but also important network bridges. Germany (16 publications; betweenness centrality = 0.18) and Australia (8 publications; betweenness centrality = 0.14) also displayed purple rings, playing key bridging roles in cross-regional collaboration. Although China ranked second in publication volume (betweenness centrality = 0.08), it did not exhibit purple rings, a phenomenon reflecting its research concentration within domestic institutional collaborations and its relatively insufficient bridging role in linking traditional dietary research with emerging frontiers such as the gut microbiome. By contrast, European countries such as Italy and Germany, despite having lower publication output than China, distributed their research across multiple topics including the Mediterranean diet, ketogenic diet, and gut microbiota, thereby exerting more prominent bridging effects. Overall, the field has formed a multipolar collaborative pattern, with the USA and China as productive cores, European countries such as Italy and Germany as knowledge hubs, and Australia as an emerging bridge. The institutional collaboration network (Figure 3b) comprised 232 nodes (*N* = 232) and 310 links (*E* = 310), with a network density of 0.0116, indicating relatively dispersed inter-institutional collaboration. The major research institutions spanned Asia, North America, and Oceania. The top 10 most productive institutions are presented in Table 1. The National Institutes of Health (NIH) (28 publications), Harvard University (26 publications), and Johns Hopkins University (9 publications) were the top three institutions, indicating that leading U.S. research institutions occupy a dominant position in this field. The remaining institutions had relatively lower publication outputs, covering universities from China, Iran, Turkey, Australia, and other countries, exhibiting a relatively balanced distribution.

**FIGURE 3:**
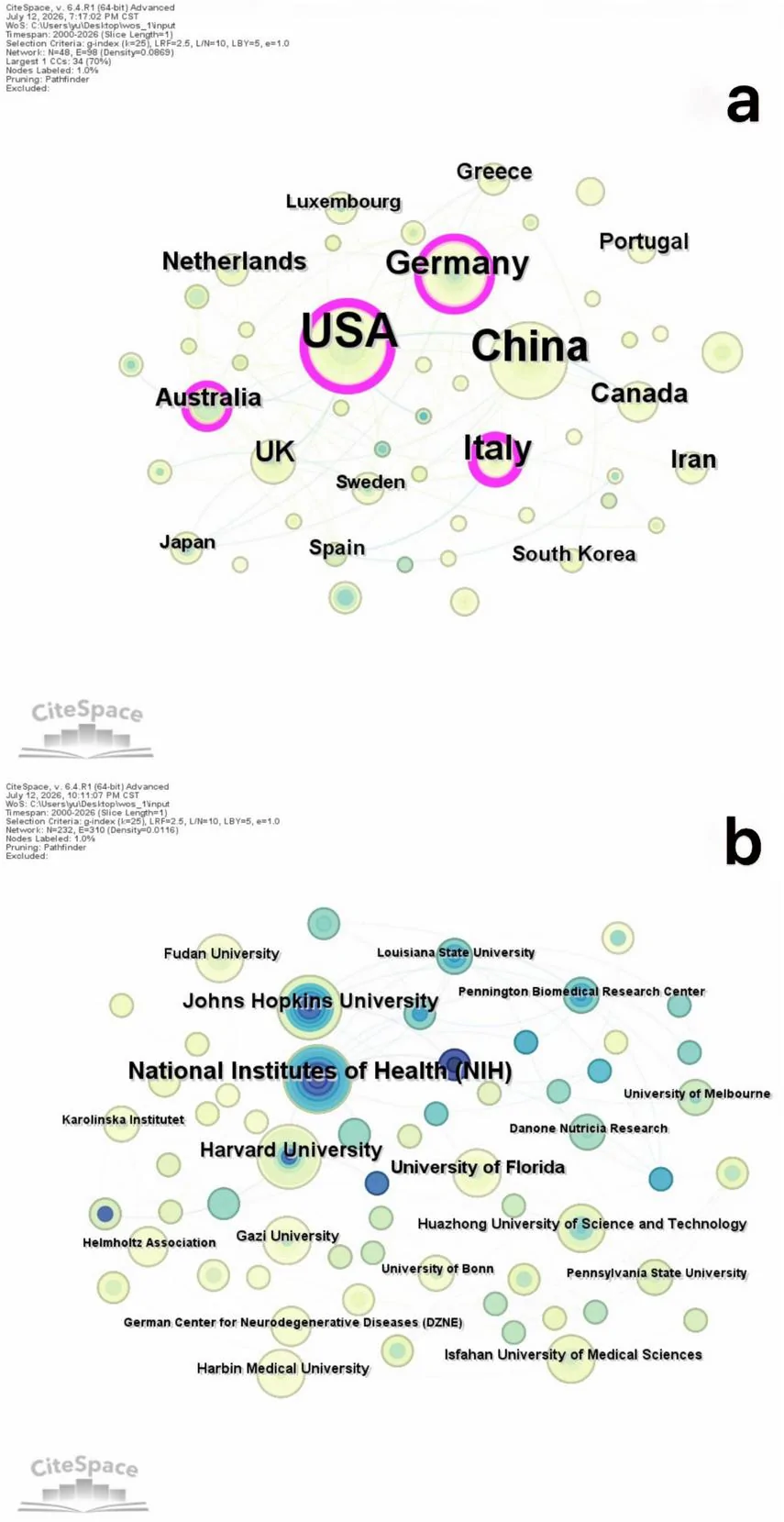
National and institutional cooperation network map: **(a)** country collaboration network; **(b)** institutional collaboration network.

**TABLE 1:** Top 10 most productive countries and institutions in research on dietary patterns for PD.

Rank	Country	Count	Citations	Rank	Institution	Count	Citations
1	USA	70	6265	1	National Institutes of Health (NIH)	28	2228
2	China	45	1244	2	Harvard University	26	2145
3	Italy	21	808	3	Johns Hopkins University	9	1113
4	Germany	16	540	4	University of Florida	6	96
5	UK	11	177	5	Gazi University	4	62
6	Poland	10	641	6	Huazhong University of Science and Technology	4	101
7	Canada	9	594	7	Isfahan University of Medical Sciences	4	172
8	Netherlands	8	315	8	Harbin Medical University	4	59
9	Iran	8	178	9	Fudan University	4	64
10	Australia	8	607	10	University of Melbourne	3	488

### 3.3 Author co-citation network and collaboration network analysis

To identify influential scholars in the field of PD dietary pattern research, an author co-citation network was constructed using CiteSpace (Figure 4a). The network comprised 1,042 nodes (*N* = 1,042) and 3,756 links (*E* = 3,756), with a network density of 0.0069. The largest connected component accounted for 91% of all nodes, indicating a high level of integration of the intellectual base in this field. The top 10 most frequently cited authors are presented in Table 2. Xiang Gao (62 citations), M. C. L. Phillips (59 citations), and Maria I. Maraki (48 citations) were the top three most cited authors, collectively constituting the primary knowledge framework of the field. Beyond citation frequency, Gao, Mattson, Bruce-Keller, and Logroscino exhibited high betweenness centrality, playing important bridging roles across different research directions. Specifically, Gao et al. (2007) revealed the epidemiological association between dietary patterns and PD risk; Mattson et al. (2003) elucidated the molecular pathways through which caloric restriction and intermittent fasting enhance neuronal resilience by inducing cellular stress responses and increasing neurotrophic factor and chaperone protein expression; Bruce-Keller et al. (2015) uncovered the mediating role of the microbiota-gut-brain axis in diet-induced neurobehavioral changes, demonstrating that gut microbiota shaped by a high-fat diet can independently induce significant alterations in exploratory behavior, cognitive performance, and stereotypic behaviors in mice in the absence of obesity; and Logroscino et al. (1996) reported a significant association between high animal fat intake and increased PD risk in a population-based case-control study. The author collaboration network (Figure 4b) comprised 402 nodes (*N* = 402) and 752 links (*E* = 752), with a network density of 0.0093, indicating relatively sparse collaborative relationships among authors. The top 10 most productive authors are presented in Table 2. Mark P. Mattson (11 publications) ranked first, followed by Alberto Ascherio (5 publications), Michela Barichella (4 publications), and Xiang Gao (3 publications). Among them, Barichella et al. (2006) focused on nutritional assessment and dietary interventions in PD patients. Notably, Mark P. Mattson, Xiang Gao, and Michela Barichella simultaneously appeared in both the high co-citation and high publication lists, indicating that they are not only founders of the intellectual base in this field but also core leaders of current research activities, with their work demonstrating both academic influence and sustained productive vitality.

**FIGURE 4:**
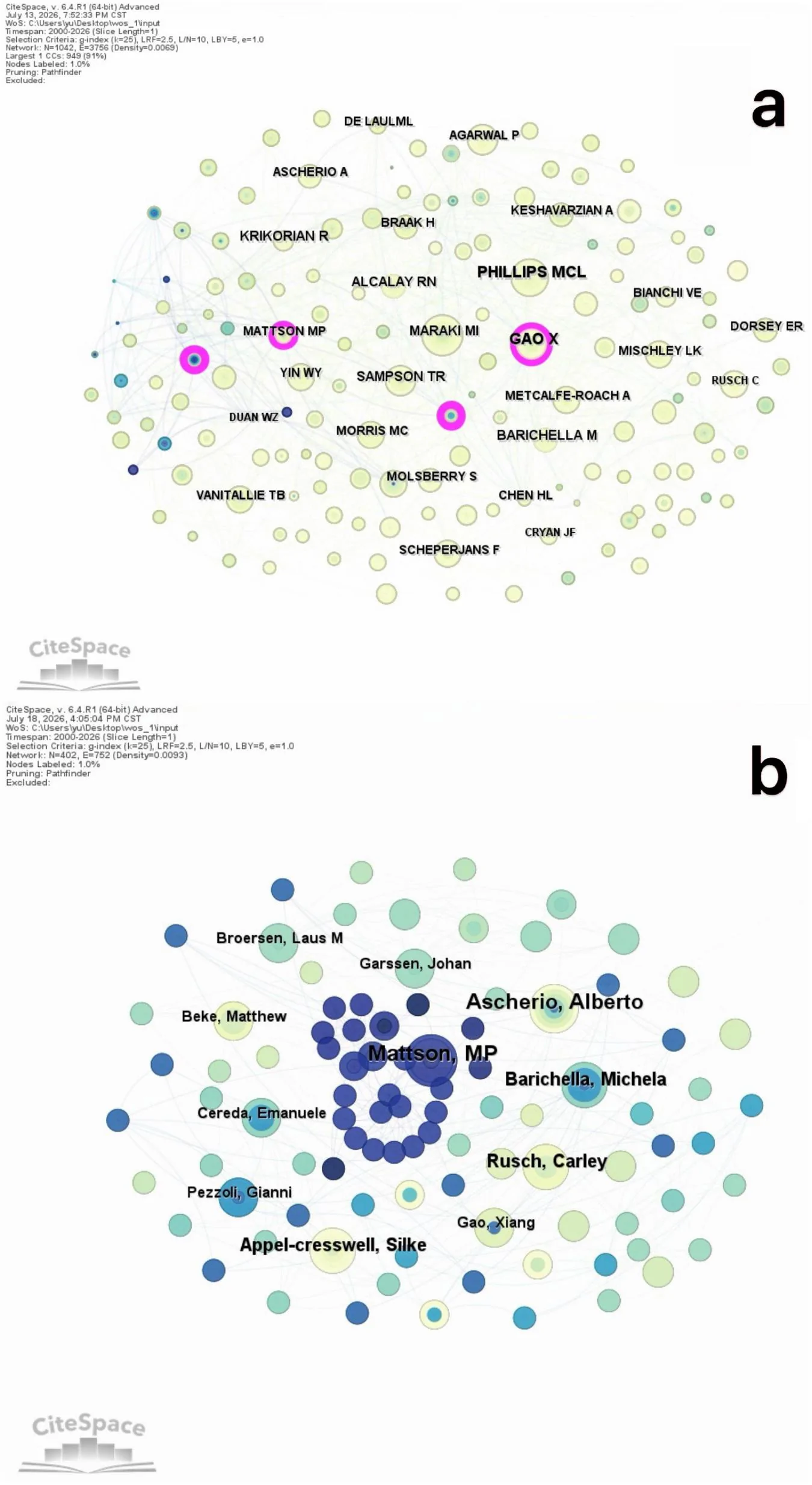
Author co-citation and collaboration network maps: **(a)** author co-citation network; **(b)** author collaboration network.

**TABLE 2:** Top 10 authors by citation count and publication count.

Rank	Cited Author	Count	Year	Author	Count	Year
1	Xiang Gao	62	2007	Mark P. Mattson	11	2000
2	M. C. L. Phillips	59	2019	Alberto Ascherio	5	2007
3	Maria I. Maraki	48	2019	Michela Barichella	4	2007
4	Roy N. Alcalay	43	2017	Carley Rusch	4	2021
5	Michela Barichella	40	2007	Silke Appel-Cresswell	4	2021
6	Rebecca Krikorian	38	2019	Johan Garssen	3	2017
7	Timothy R. Sampson	36	2019	Emanuele Cereda	3	2010
8	Pankaj Agarwal	33	2021	Xiang Gao	3	2007
9	Martha Clare Morris	32	2017	Gianni Pezzoli	3	2007
10	Theodore B. VanItallie	32	2014	Matthew Beke	3	2021

### 3.4 Journal dual-map overlay

Dual-map overlay analysis can reveal the disciplinary positioning and knowledge evolution pathways of a research field. As shown in Figure 5, the left side represents citing journals, the right side represents cited journals, and the curved lines denote citation paths, with thickness reflecting the strength of knowledge flow. The field takes the “MOLECULAR, BIOLOGY, GENETICS” cluster on the right as its core knowledge foundation, with major citation flow directed toward the “MOLECULAR, BIOLOGY, IMMUNOLOGY” cluster on the left, indicating that research is shifting from molecular genetic mechanisms toward immunomodulatory directions, reflecting a trend of translating fundamental discoveries toward clinical applications. In summary, PD dietary pattern research exhibits interdisciplinary characteristics, with molecular genetics as its foundation, immunomodulation as its frontier, and clinical translation as its orienting goal.

**FIGURE 5:**
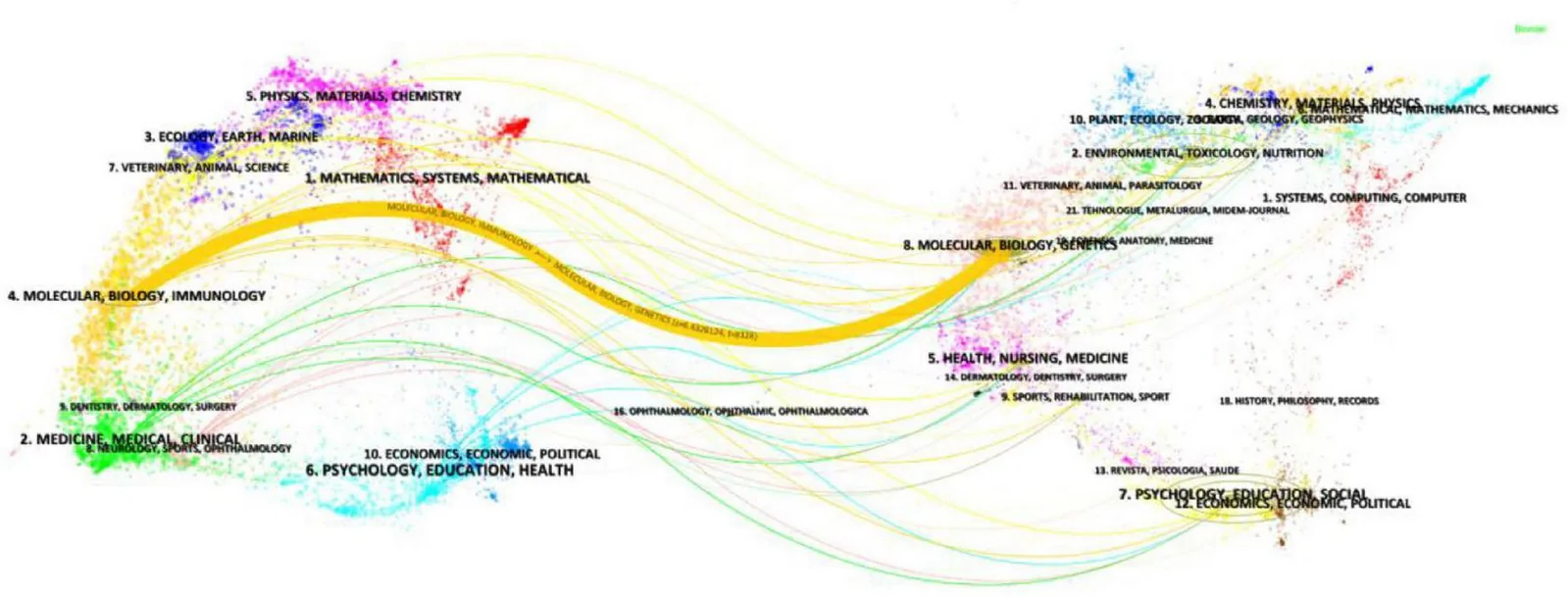
Journal dual-map overlay.

### 3.5 Reference analysis

#### 3.5.1 Co-citation network and cluster map

Reference co-citation analysis can identify the intellectual base of the PD dietary pattern research field. A reference co-citation network was constructed using CiteSpace (Figure 6a). The network comprised 1,066 nodes (*N* = 1,066) and 3,176 links (*E* = 3,176), with a network density of 0.0056. The largest connected component included 415 nodes (38%), indicating a moderate degree of concentration in the core literature cluster and relatively extensive co-citation associations among high-impact publications. Based on the co-citation network, cluster analysis was performed using the log-likelihood ratio (LLR) algorithm to extract cluster labels, resulting in the formation of four major clusters (Figure 6b). The quality of the cluster map can be assessed by the Modularity Q value and the Mean Silhouette S value. A Q value > 0.3 indicates that the clustering structure is significantly reasonable, while an S value ≥ 0.7 indicates that the clustering results are reliable. The cluster map yielded *Q* = 0.8327 and *S* = 0.9857, indicating a significant clustering structure with extremely high internal homogeneity and clearly defined boundaries among the knowledge domains. Brain aging (#0), as a core cluster, focuses on the shared mechanisms between brain aging and neurodegeneration, which represent important targets for PD dietary interventions. Anti-aging dietary strategies such as caloric restriction and intermittent fasting exert neuroprotective effects by delaying the brain aging process (Lobo et al., 2022). Nigrostriatal degeneration (#2), representing the core pathological feature of PD, focuses on the degeneration of nigral dopaminergic neurons. Studies have shown (Tosefsky et al., 2024) that dietary patterns may protect this pathway through antioxidant and anti-inflammatory mechanisms, making it an important target for dietary intervention research. The 3-nitropropionic acid (#1) cluster focuses on mitochondrial dysfunction and energy metabolism. This mitochondrial complex II inhibitor is commonly used to model metabolic disturbances in PD, reflecting the preclinical research foundation of metabolic intervention strategies such as the ketogenic diet and caloric restriction (Guo et al., 2000). The Parkinson’s disease (#3) cluster serves as a foundational cluster, bringing together fundamental literature on PD research and representing the broadest knowledge framework in this field.

**FIGURE 6:**
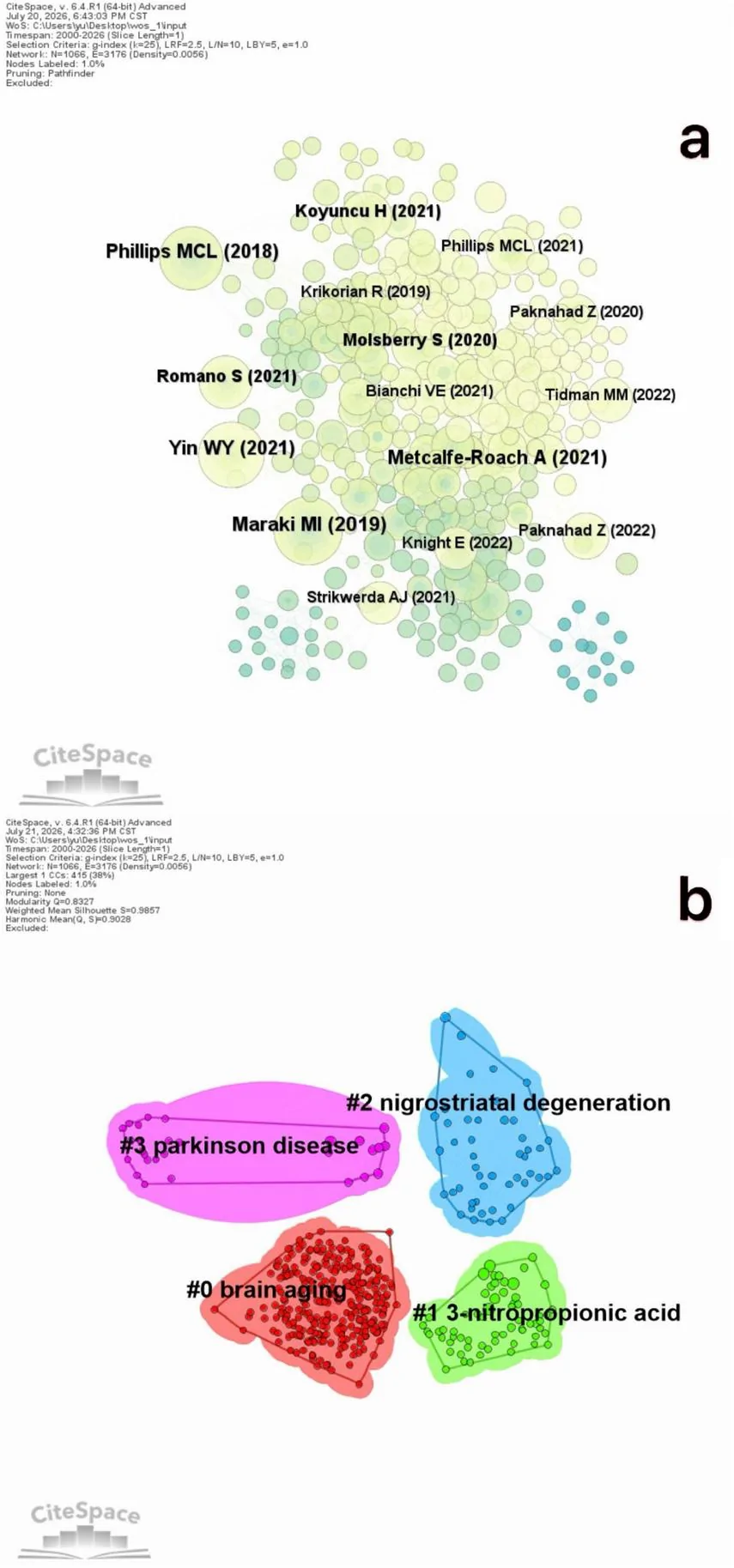
Co-citation network and cluster map of cited references: **(a)** co-citation network; **(b)** cluster map.

#### 3.5.2 Highly co-cited representative references

Citation frequency serves as an important indicator of a publication‘s contribution to the field, and the top 10 references ranked by co-citation frequency are presented in Table 3. These publications encompass a complete research chain extending from epidemiological associations between dietary patterns and disease risk, through randomized controlled trials of metabolic interventions, to investigations into gut microbiota mechanisms. Among the 10 publications, 7 were published in Q1 journals, with Movement Disorders contributing 4 articles, and Neurology and npj Parkinson’s disease each contributing 1 article, reflecting the relatively high academic impact of research output in this field. At the epidemiological level, the study by Maraki et al. (2019), which ranked first in citation frequency, represents core evidence for the association between the Mediterranean diet and prodromal PD. Yin et al. (2021), based on a Swedish national cohort, further supported the protective role of the Mediterranean diet in PD prevention. Molsberry et al. (2020) demonstrated that adherence to a healthy dietary pattern may reduce the non-motor symptoms that frequently precede a PD diagnosis. In addition, Metcalfe-Roach et al. (2021) provided important evidence linking the MIND diet to delayed onset of PD. At the level of dietary pattern comparison and metabolic intervention, Phillips et al. (2018), through a preliminary randomized controlled trial, confirmed that a ketogenic diet was superior to a low-fat diet in improving non-motor symptoms in PD patients, providing initial evidence for metabolic intervention strategies. Koyuncu et al. (2021) validated the improving effect of a ketogenic diet on voice quality in PD patients, while Krikorian et al. (2019) demonstrated the potential of a ketogenic diet to improve cognitive function in PD patients with mild cognitive impairment. At the mechanistic level, Romano et al. (2021), through a meta-analysis, systematically summarized the consistent alterations in the gut microbiota of PD patients, revealing a reduction in short-chain fatty acid-producing bacteria and enrichment of pro-inflammatory bacteria. This provided evidence for the involvement of gut dysbiosis in PD pathogenesis and offered mechanistic clues for understanding how dietary patterns may influence PD through the gut microbiota. Collectively, these publications, spanning disease prevention, prognostic improvement, metabolic intervention, and mechanistic exploration, constitute a multidimensional knowledge base in this field.

**TABLE 3:** Top 10 references ranked by co-citation frequency.

Rank	Cited frequency	Title	First author	Journal	Year
1	31	Mediterranean diet adherence is related to reduced probability of prodromal Parkinson’s disease	Maria I. Maraki	Movement Disorders (7.7,Q1)	2019
2	28	Mediterranean dietary pattern at middle age and risk of Parkinson’s disease: a Swedish cohort study	Weiyao Yin	Movement Disorders (7.7,Q1)	2021
3	27	Low-fat versus ketogenic diet in Parkinson’s disease: a pilot randomized controlled trial	Matthew C.L. Phillips	Movement Disorders (7.7,Q1)	2018
4	26	MIND and Mediterranean diets associated with later onset of Parkinson’s disease	Avril Metcalfe-Roach	Movement Disorders (7.7,Q1)	2021
5	19	Diet pattern and prodromal features of Parkinson disease	Samantha Molsberry	Neurology (8.9,Q1)	2020
6	19	Meta-analysis of the Parkinson’s disease gut microbiome suggests alterations linked to intestinal inflammation	Stefano Romano	npj Parkinson’s Disease (8.5,Q1)	2021
7	16	Effect of ketogenic diet versus regular diet on voice quality of patients with Parkinson’s disease	Handan Koyuncu	Acta Neurologica Belgica (2.0,Q3)	2021
8	15	Randomized crossover trial of a modified ketogenic diet in Alzheimer’s disease	Matthew C.L. Phillips	Alzheimer’s Research & Therapy (8.9,Q1)	2021
9	15	The effects of Mediterranean diet on severity of disease and serum total antioxidant capacity (TAC) in patients with Parkinson’s disease: a single center, randomized controlled trial	Zamzam Paknahad	Nutritional Neuroscience (3.1,Q2)	2022
10	13	Nutritional ketosis for mild cognitive impairment in Parkinson’s disease: A controlled pilot trial	Robert Krikorian	Clinical Parkinsonism and Related Disorders (2.7,Q3)	2019

### 3.6 Keyword analysis

#### 3.6.1 Keyword co-occurrence, clustering, and timeline visualization

Keyword co-occurrence network analysis can reveal the hot topics, knowledge structure, and evolutionary trajectories of a research field. A keyword co-occurrence network for PD dietary pattern research was constructed using CiteSpace (Figure 7a). The network comprised 525 nodes (*N* = 525) and 1,643 links (*E* = 1,643), with a network density of 0.0119. The largest connected component included 469 nodes, accounting for 89% of all nodes, indicating that keyword co-occurrence relationships are relatively concentrated and that core research themes form a tightly interconnected network. The top 15 high-frequency keywords are presented in Table 4.

**FIGURE 7:**
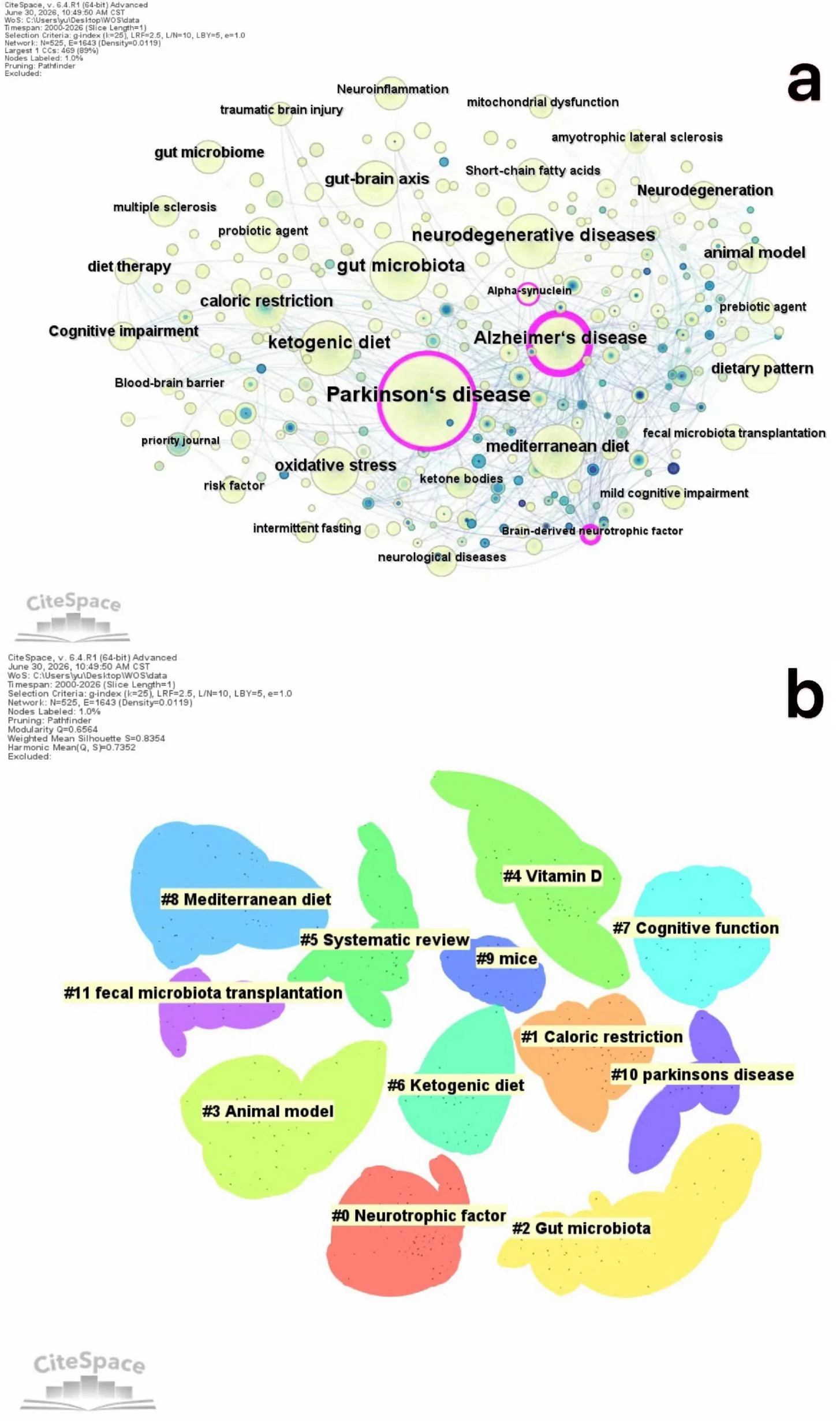
Keyword co-occurrence network and cluster map: **(a)** keyword co-occurrence network; **(b)** keyword cluster map.

**TABLE 4:** Top 15 high-frequency keywords in research on dietary patterns for PD.

Rank	Keyword	Count	Centrality	Year
1	Parkinson’s disease	255	0.15	2002
2	Ketogenic diet	107	0.04	2003
3	Alzheimer’s disease	104	0.25	2002
4	Neurodegenerative diseases	97	0.01	2004
5	Gut microbiota	92	0	2018
6	Mediterranean diet	89	0.01	2009
7	Oxidative stress	87	0.02	2002
8	Caloric restriction	81	0.1	2002
9	Gut-brain axis	57	0	2019
10	Animal model	47	0.05	2010
11	Cognitive impairment	41	0.07	2009
12	Diet therapy	38	0.11	2009
13	Gut microbiome	37	0.01	2018
14	Dietary pattern	36	0.02	2007
15	Neurodegeneration	34	0.1	2011

From the distribution of high-frequency keywords, four major themes can be identified. In the disease and target theme, “Parkinson’s Disease” exhibited the highest frequency and was marked with a purple ring (betweenness centrality = 0.15), serving as a core hub node in the knowledge network of this field. “Alzheimer’s disease” also displayed a purple ring (betweenness centrality = 0.25), indicating its important bridging role within the knowledge network. Its higher betweenness centrality than PD suggests that dietary research in PD may be informed by neuroprotective paradigms extensively investigated in AD research, reflecting shared dietary and mechanistic frameworks across neurodegenerative diseases (Ayten and Bilici, 2024). “Alpha-synuclein” likewise featured a purple ring, positioned at the intersection of disease and mechanism themes, and its high betweenness centrality indicates that α-synuclein pathology serves as a key bridge connecting dietary interventions with PD pathological processes. In addition, “cognitive impairment” suggests that improving PD-related cognitive impairment through dietary interventions represents an important current research direction. In the dietary pattern and intervention theme, the emergence of high-frequency keywords such as “ketogenic diet,” “mediterranean diet,” “caloric restriction,” and “ketone bodies” indicates that metabolic intervention strategies occupy a central position in PD dietary pattern research. “Dietary pattern,” as a summarizing node, reflects the shift in research focus from single nutrients to overall dietary patterns, while “diet therapy” suggests that the clinical translation value of dietary interventions continues to attract attention. In the mechanism and model theme, “oxidative stress,” as a core element of PD pathogenesis, reflects the close association between oxidative stress and neurodegeneration. “Neurodegeneration” and “neuroinflammation” represent dual regulatory targets of neurodegeneration and neuroinflammation in dietary interventions. “Animal model” indicates the foundational role of preclinical experimental validation systems, while “mitochondrial dysfunction” suggests that mitochondrial function serves as an energy metabolism target for dietary interventions. Notably, “brain-derived neurotrophic factor” (centrality = 0.13) was marked with a purple ring, suggesting its bridging role in connecting dietary interventions with neuroplasticity improvement (Alkurd et al., 2024). In the gut microbiota regulation theme, “gut microbiota,” as a core node, is directly connected to “gut-brain axis,” forming the core framework of the “microbiota-gut-brain” regulatory network. Intervention nodes such as “probiotic agent,” “prebiotic agent,” and “fecal microbiota transplantation” are distributed alongside metabolic markers such as “short-chain fatty acids,” indicating that emerging intervention modalities including probiotics, prebiotics, fecal microbiota transplantation, and gut microbial metabolites represent important frontier directions in PD dietary pattern research.

On this basis, cluster analysis was performed on the keyword co-occurrence network using the LLR algorithm, identifying 12 major clusters (Figure 7b). The clustering map yielded *Q* = 0.6564 (>0.3) and *S* = 0.8354 (>0.7), indicating a significant clustering structure with good internal homogeneity and clearly defined boundaries among knowledge modules. These clustering results were highly consistent with the four major themes identified above. In the disease and target theme, Parkinson’s disease (#10) served as the core, while cognitive function (#7) focused on cognitive impairment improvement. The dietary pattern and intervention theme encompassed caloric restriction (#1), ketogenic diet (#6), Mediterranean diet (#8), and vitamin D (#4). The mechanism and model theme included neurotrophic factor (#0), animal model (#3), and mice (#9). The gut microbiota regulation theme consisted of gut microbiota (#2) and fecal microbiota transplantation (#11). In addition, systematic review (#5) emerged as an independent methodological cluster. In terms of cluster size, the gut microbiota regulation cluster represented by gut microbiota (#2) and fecal microbiota transplantation (#11), together with the metabolic intervention strategy cluster constituted by caloric restriction (#1) and ketogenic diet (#6), were the two largest clusters, jointly forming the core knowledge domains of PD dietary pattern research. The timeline view (Figure 8) further illustrated that the research field has progressively expanded from early single-nutrient studies to metabolic intervention strategies and further extended to gut microbiota regulation.

**FIGURE 8:**
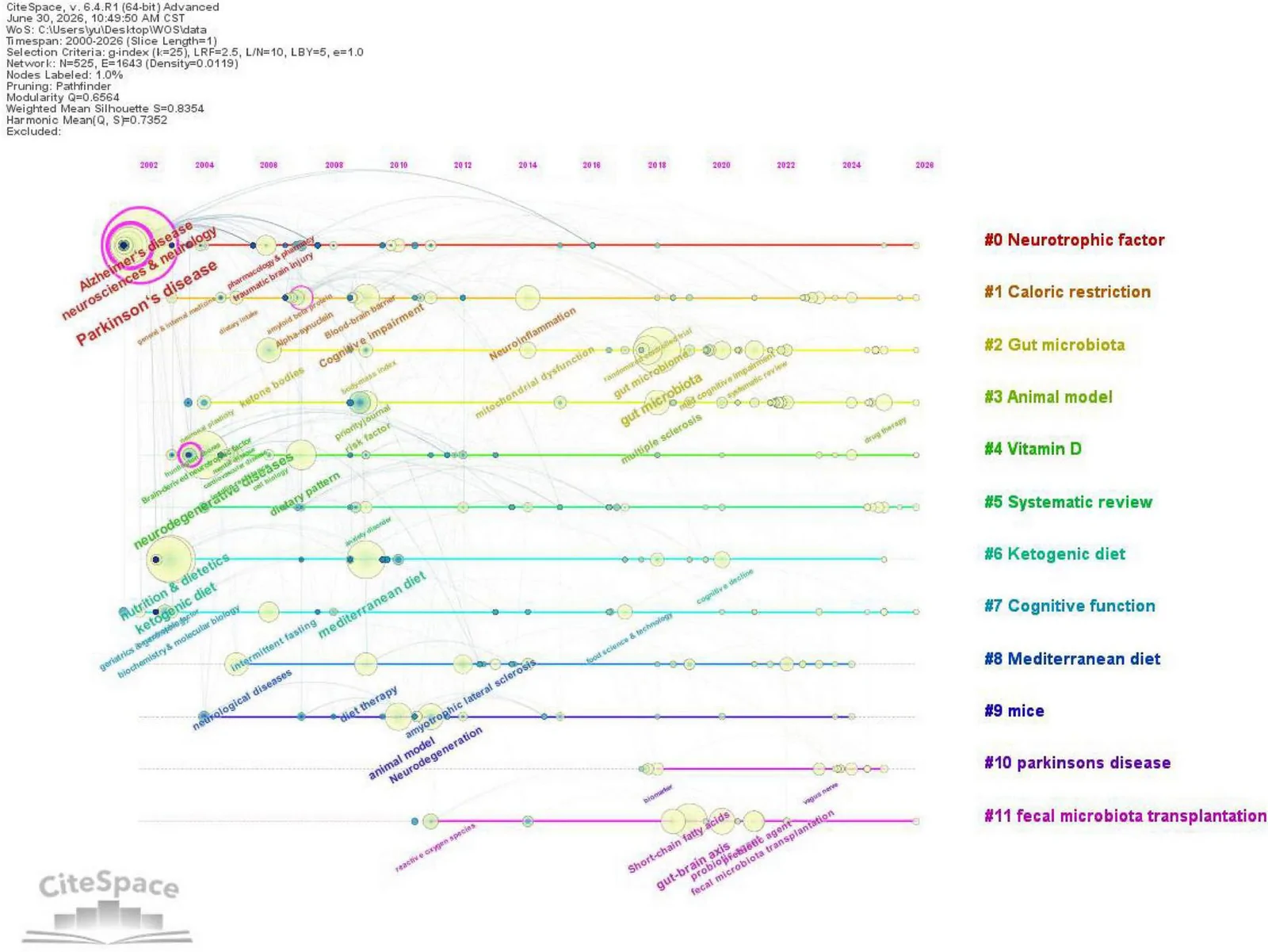
Keyword timeline view.

#### 3.6.2 Keyword burst detection

Keyword burst detection can identify research hotspots with sharply increased citation frequency during specific time periods. Based on the CiteSpace algorithm, a total of 23 high-intensity burst keywords were identified (Figure 9). The results showed that research from 2002 to 2016 was predominantly focused on basic mechanistic exploration. “Caloric restriction” (strength = 9.94) sustained its burst for 14 years, establishing the foundational role of metabolic intervention. Keywords such as “antioxidant activity” and “neurotrophic factor” reflected early attention to fundamental mechanisms including antioxidant and neurotrophic support. The period from 2018 to 2021 exhibited characteristics of clinical translation transition, with “ketone bodies” suggesting the translation of metabolic intervention strategies from basic research toward clinical application, and the burst of “mild cognitive impairment” indicating growing interest in the potential value of dietary interventions for early cognitive protection in PD. From 2022 to 2026, the gut microbiota and overall dietary patterns emerged as explosive frontiers. “Gut-brain axis” (strength = 3.82) and “dietary pattern” (strength = 3.66) were the strongest burst keywords, indicating a shift in research focus toward the integrated “microbiota-gut-brain axis” system and overall dietary framework. The concentrated bursts of “probiotic agent,” “prebiotic agent,” and “fecal microbiota transplantation” further indicated that gut microbiota intervention strategies are becoming an important frontier direction in this field.

**FIGURE 9:**
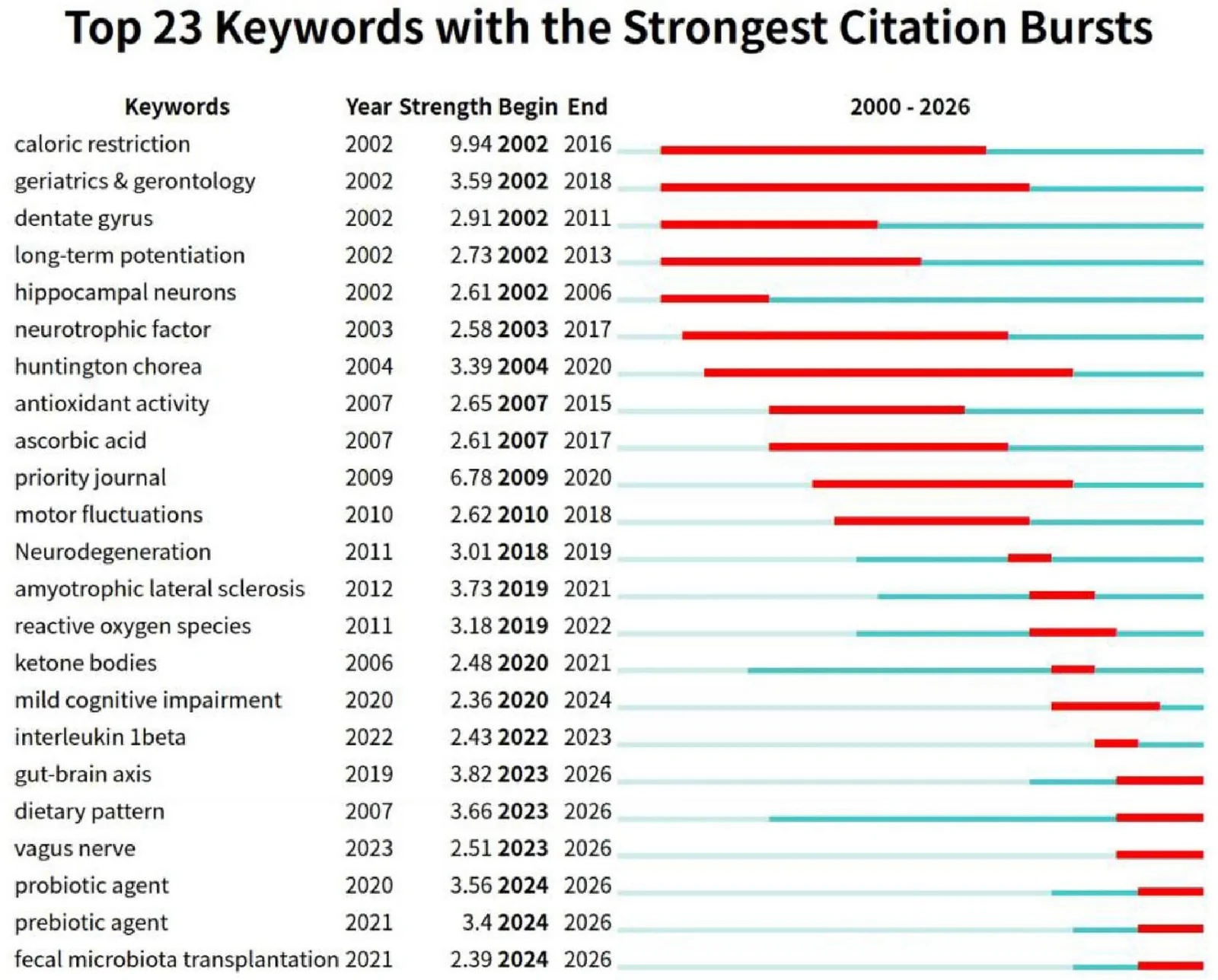
Keyword burst map.

## 4 Discussion

### 4.1 Overall development trends

This study systematically mapped the development trajectory of PD dietary pattern research. The annual publication output showed a fluctuating upward trend, with a cumulative total of 424 publications. After 2022, the average annual output increased markedly, indicating that the field has transitioned from early exploration into a phase of rapid growth. At the country level, the USA and China constituted the productive cores, while Italy and Germany formed a European research cluster. Notably, the USA, Italy, Germany, and Australia exhibited high betweenness centrality, playing bridging roles in cross-regional collaborations. At the institutional level, the NIH, Harvard University, and Johns Hopkins University ranked among the top contributors, all representing leading U.S. research institutions, while universities from China, Iran, Turkey, and Australia also entered the top ten, reflecting a multipolar diffusion of research capacity. Among the core authors, Mark P. Mattson ranked first in publication output, focusing on the neuroprotective mechanisms of caloric restriction and intermittent fasting, whereas Alberto Ascherio and Xiang Gao concentrated on population-based associations between dietary patterns and PD risk. Notably, seven of the highly co-cited publications were published in Q1 journals, reflecting the relatively high academic impact of this field and the establishment of a diverse knowledge base centered on epidemiology, metabolic intervention, and gut microbiota.

### 4.2 Research hotspots and emerging trends

Keyword burst detection has revealed a frontier shift in PD dietary pattern research. The recent burst of “dietary pattern” indicates that the field is transitioning from “single nutrients” toward an “overall dietary framework.” Romano et al. (2021), through a meta-analysis, systematically summarized the consistent alterations in the gut microbiota of PD patients, identifying a reduction in short-chain fatty acid-producing bacteria and an enrichment of pro-inflammatory bacteria. This suggests that dietary patterns may influence PD pathogenesis by modulating gut microbial composition and their metabolites. Single-nutrient studies are insufficient to explain the overall effects of dietary interventions, because different dietary components can undergo synergistic metabolism and cross-feeding at the gut microbiota level (Piccioni et al., 2023), and their combined effects cannot be linearly predicted from the effects of individual components (Choi et al., 2022). This provides a critical basis for establishing “dietary pattern” as an overall dietary framework.

The simultaneous bursts of “gut-brain axis” and “vagus nerve” indicate that current research is focusing on the verification of specific communication pathways in gut-brain signaling. The vagus nerve, as the main peripheral afferent pathway of the gut-brain axis, constitutes the anatomical basis for the transmission of intestinal signals to the central nervous system (Mendez-Hernandez et al., 2025). Margolis et al. (2021) systematically elucidated the role of the microbiota-gut-brain axis in regulating gut and central nervous system functions, clearly indicating that intestinal signals can be transmitted to the brain via vagal afferent pathways. On this basis, the burst of “interleukin 1beta” further revealed the inflammatory regulatory mechanisms of this pathway at the molecular level. IL-1β is a key cytokine that initiates and amplifies neuroinflammatory cascades (Marinescu et al., 2026). Gut dysbiosis may promote the production and release of pro-inflammatory mediators such as IL-1β through activation of the intestinal innate immune system (Sadowski et al., 2024). These inflammatory signals may influence central nervous system function through vagal afferent and circulatory pathways. Taken together, these findings suggest that the gut-brain axis, vagal signaling, and IL-1β-mediated inflammation may represent interconnected mechanisms relevant to PD; however, whether specific dietary patterns directly modulate this pathway in humans remains to be established.

Gut microbiota intervention strategies have emerged as a frontier direction in PD dietary pattern research. The concurrent bursts of “probiotic agent,” “prebiotic agent,” and “fecal microbiota transplantation” indicate that research on gut microbiota modulation has expanded from observational studies of dietary associations toward interventional evaluation. In the PD field, clinical evidence for probiotics has been preliminarily accumulated. A meta-analysis by Park et al. (2023) showed that probiotic supplementation could improve motor symptoms, non-motor symptoms, and depression scores in PD patients, but did not show significant improvements in endpoints such as constipation or antioxidant capacity. Barichella et al. (2016) found that fermented milk containing multi-strain probiotics could relieve constipation symptoms in PD patients. However, studies in this area generally have small sample sizes, short intervention durations, and considerable variability in bacterial strains and dosages, so no standardized regimen has yet been established for generalization. Compared with probiotics, the clinical evidence for FMT and prebiotics is weaker. The randomized controlled trial of FMT conducted by Scheperjans et al. (2024) showed that although FMT was acceptably safe, it did not demonstrate superior clinical improvement over placebo in primary motor endpoints. Evidence for prebiotics is equally limited, but studies have begun to explore their independent intervention value. Hegelmaier et al. (2026) enrolled 72 PD patients and administered short-chain fatty acids (SCFAs), the prebiotic 2′-fucosyllactose, or a combination of both for 6 months; the combined supplementation showed the most significant benefits in improving stool consistency, defecation frequency, and constipation-related quality of life. Collectively, these findings indicate that although research has progressed from observational associations toward direct interventional testing of microbiota-targeted strategies, the available clinical findings remain heterogeneous and do not yet provide unequivocal evidence of therapeutic efficacy in PD. Further well-designed clinical trials are therefore needed.

The burst of “mild cognitive impairment” reflects the refining focus of PD dietary pattern research on cognitive endpoints. Early studies often used the broad term “cognitive impairment” to describe cognitive decline. With the maturation of the concept of mild cognitive impairment staging, “mild cognitive impairment” has entered the research landscape as a more specific clinical endpoint. A controlled trial found that a ketogenic diet could improve word retrieval and memory performance in PD patients with mild cognitive impairment (Krikorian et al., 2019). Complementary evidence from a pilot randomized controlled trial showed that the ketogenic diet produced a greater improvement in MDS-UPDRS Part I than the low-fat diet, with cognitive impairment among the individual non-motor items showing a greater between-group decrease (Phillips et al., 2018). However, dietary intervention evidence specifically targeting PD-MCI remains very limited, as most studies have concentrated on motor symptoms or disease risk.

Taken together, these burst keywords collectively depict the overall landscape of PD dietary pattern research. Dietary interventions have formed a clear pathway, starting from overall dietary exposure, proceeding through gut microbiota modulation, and ultimately leading to improved clinical outcomes. Current research is at a critical transitional stage from mechanistic validation to clinical translation, and future studies should further explore key issues including standardized intervention protocols, population stratification strategies, and long-term follow-up validation.

### 4.3 Research gaps and future directions

Through bibliometric analysis, this study has revealed the knowledge structure and evolutionary trends in PD dietary pattern research, while also identifying several notable research gaps. The most prominent limitation in this field is that the current evidence base is predominantly composed of observational studies, with a marked scarcity of high-quality randomized controlled trials. Multiple systematic reviews and meta-analyses have consistently indicated that observational studies overwhelmingly dominate the existing literature, and the causal relationship between dietary patterns and PD has not yet been established (Zhang et al., 2025; Zhao et al., 2025). Furthermore, the definition and assessment methods for dietary patterns vary considerably across studies, making direct comparisons of results difficult and insufficient to support the formulation of clinical dietary guidelines (Knight et al., 2022).

For different dietary patterns, there are notable evidence gaps in the current research. For example, regarding the Mediterranean diet, existing evidence suggests potential benefits for cognitive function and gastrointestinal symptoms, but its effects on motor symptoms remain unclear (Seelarbokus et al., 2024). For the ketogenic diet, small-scale clinical trials have suggested possible improvements in non-motor symptoms, but sample sizes are generally small, intervention durations are short, and long-term safety and tolerability have not been systematically evaluated (Choi et al., 2024). Regarding the MIND diet, the evidence for delayed PD onset initially came from cross-sectional studies. A recent prospective cohort study (Hajji-Louati et al., 2026) found that the association between the MIND diet and PD incidence was only significant in women under 71 years of age, with no significant association observed in the overall population, indicating that these findings require further validation. For protein-modified diets, current evidence is insufficient to support low-protein diets, whereas protein redistribution has been associated with improved motor function (Cereda et al., 2010). Nevertheless, high-level evidence for both low-protein and protein-redistribution diets remains limited (Rusch et al., 2023). Plant-based dietary approaches represent another emerging area of interest in PD research; however, clinical evidence remains limited, particularly regarding intervention studies in patients with established PD, highlighting the need for further well-designed clinical trials (Tosefsky et al., 2024). Moreover, comparative studies between different dietary patterns are extremely scarce, and it remains unclear which dietary pattern is most advantageous for which symptoms at which disease stage. At the population level, the protective effect of the Mediterranean diet on prodromal PD appears to be significantly stronger than on established PD, but no dietary RCT specifically targeting prodromal PD or MCI populations has been reported (Zhao et al., 2025). At the mechanistic level, dietary patterns have been associated with alterations in gut microbiota composition and short-chain fatty acid production, but current evidence cannot distinguish causal associations from correlational relationships, and the complete causal chain has not yet been validated in humans (Kwon et al., 2024; Seelarbokus et al., 2024).

In summary, PD dietary pattern research is currently at a critical transitional stage from mechanistic validation to clinical translation. Future studies should prioritize well-designed, large-scale randomized controlled trials with standardized intervention protocols to establish causal evidence between dietary interventions and clinical outcomes. Second, the intervention window should be shifted forward, with a focus on early dietary interventions in prodromal PD and PD patients with mild cognitive impairment. Concurrently, further mechanistic investigations are needed to deepen the understanding of the causal chain along the microbiota-gut-brain axis, clarifying the key pathways through which dietary components influence neurological function via gut microbial metabolism, thereby providing a biological basis for precision dietary intervention strategies. Figure 10 provides a schematic overview of the current evidence, identified research gaps, and potential future directions toward clinical translation in PD dietary research.

**FIGURE 10:**
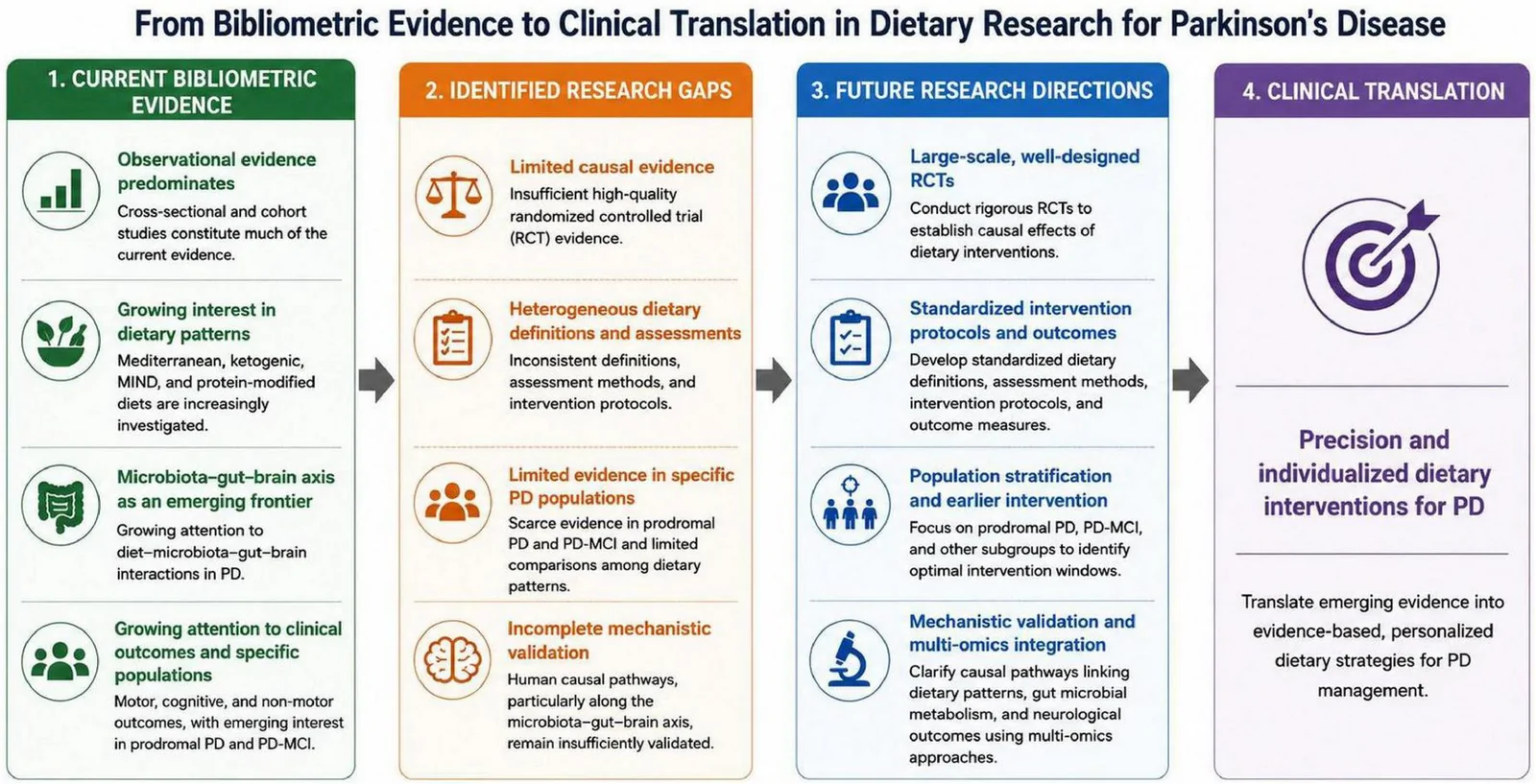
Schematic overview of clinical translation in PD dietary research.

## 5 Limitations

This study has several limitations. First, although WoSCC and Scopus are widely used in bibliometric research, the exclusion of biomedical databases such as PubMed/MEDLINE and Embase may have resulted in the omission of some relevant publications, particularly those retrievable through database-specific controlled vocabulary. This potential database coverage bias should be considered when interpreting the findings. In addition, the inclusion of only English-language publications may have introduced language bias. Second, citation-based indicators may be influenced by publication age, visibility, author prominence, and disciplinary or regional citation practices and therefore should not be interpreted as direct measures of scientific quality or clinical impact. Third, bibliometric analysis does not assess the methodological rigor or clinical reliability of individual studies, while thematic clustering and burst detection may be influenced by algorithm and parameter selection, and changes in terminology over time may not be fully captured by keyword-based analyses. Fourth, the publication data for 2026 were incomplete at the time of analysis, and the findings represent a time-specific snapshot of this rapidly evolving field. Finally, bibliometric analysis complements rather than replaces clinical evidence synthesis. Given the heterogeneity of the underlying literature and the limited number of high-quality trials, the identified hotspots and emerging trends should be interpreted as patterns of research activity rather than as direct evidence of clinical efficacy.

## 6 Conclusion

This study presents the first systematic bibliometric analysis of the research landscape on dietary patterns in Parkinson‘s disease, revealing the knowledge structure, evolutionary trajectories, and frontier transitions in this field. The findings indicate that the field has progressively expanded from early explorations of caloric restriction and antioxidant mechanisms toward an immuno-metabolic regulatory network centered on the microbiota-gut-brain axis, with overall dietary patterns replacing single nutrients as the dominant analytical framework. However, the current evidence base remains predominantly observational, and the causal relationship between dietary interventions and clinical outcomes in PD has yet to be established. Therefore, no specific dietary pattern can currently be recommended with confidence for the clinical management of PD (Trinh et al., 2026). Future efforts should prioritize high-quality randomized controlled trials to establish causal evidence, advance the intervention window to prodromal and early-stage PD populations, and leverage multi-omics technologies to further investigate the mechanisms underlying microbiota-gut-brain interactions, thereby promoting dietary patterns from adjunctive management strategies toward precision and individualized clinical practice.

## Data Availability

The original contributions presented in this study are included in this article/Supplementary material, further inquiries can be directed to the corresponding authors.

## Footnotes

1
https://osf.io/vgc9p
