# Supplementary material for: Bibliometric analysis of dietary patterns in Parkinson’s disease: from Mediterranean diet to emerging trends
Source: Front Aging Neurosci. 2026 Sep 23;18:1954819. doi: 10.3389/fnagi.2026.1954819 (PMC13642293; doi:10.3389/fnagi.2026.1954819)
Supplement: Supplementary file 1 [file Table_1.docx]

| Data sources | Search | Query | Results |
| --- | --- | --- | --- |
| Web of Science | **#1** | TS=("Parkinson*" OR "Parkinson's disease") | 252,945 |
|  | **#2** | TS=( "Mediterranean diet" OR "ketogenic diet" OR "MIND diet" OR "plant-based diet" OR "vegetarian diet" OR "vegan diet" OR "DASH diet" OR "low-protein diet" OR "intermittent fasting" OR "low-fat diet" OR "caloric restriction" OR "Paleolithic diet" OR "paleo diet" OR "dietary pattern*" OR "diet* intervention" OR "whole diet" OR "nutrition* pattern*") | 86,066 |
|  | **#3** | 1# AND 2# | 695 |
| Scopus | **#1** | TITLE-ABS-KEY ( "Parkinson*" OR "Parkinson's disease" ) | 269,579 |
|  | **#2** | TITLE-ABS-KEY ( "Mediterranean diet" OR "ketogenic diet" OR "MIND diet" OR "plant-based diet" OR "vegetarian diet" OR "vegan diet" OR "DASH diet" OR "low-protein diet" OR "intermittent fasting" OR "low-fat diet" OR "caloric restriction" OR "Paleolithic diet" OR "paleo diet" OR "dietary pattern*" OR "diet* intervention" OR "whole diet" OR "nutrition* pattern*" ) | 125,107 |
|  | **#3** | 1# AND 2# | 1,102 |

**Appendix A: Search Strategy**
